# Capillary Electrophoresis-Laser Induced Fluorescence Method Development and Validation for Quantification of Nine Gangliosides—Application to Analysis of Cell Lines of CNS Origin

**DOI:** 10.3390/molecules29163769

**Published:** 2024-08-09

**Authors:** Katinka Tarnóczi, Orsolya Geda, Tamás Tábi, Éva Szökő

**Affiliations:** Department of Pharmacodynamics, Semmelweis University, 4 Nagyvárad tér, H-1089 Budapest, Hungary; tarnoczi.katinka.timea@semmelweis.hu (K.T.); geda.orsolya@semmelweis.hu (O.G.); szoko.eva@semmelweis.hu (É.S.)

**Keywords:** gangliosides, capillary electrophoresis, method validation, CNS origin cell lines

## Abstract

Gangliosides are sialic acid-containing glycosphingolipids that play an essential role in many biological and pathophysiological processes. They are present in high amounts in the central nervous system and their abnormal metabolism or expression has been observed in many diseases. We have developed and validated a sensitive capillary electrophoresis laser-induced fluorescence (CE-LIF) method for the separation and quantification of oligosaccharides digested from nine gangliosides of high biological relevance. APTS was used for the labeling of the glycans. Reverse polarity CE was performed for the separation of the labeled glycans bearing negative charges. The optimized background electrolyte is a 15 mM lithium acetate buffer with pH of 5 containing 5% *w*/*v* linear polyacrylamide, which allows for the separation of all nine gangliosides. Validation parameters including linearity, precision, and accuracy were evaluated. LOQ and LOD were in the nM range, comparable to those of LC-MS techniques. The method was used to identify and quantify the ganglioside pattern of glioblastoma and neuroblastoma cell lines. The presented method is a valuable tool for further investigations aiming at understanding the role of gangliosides in various neurological diseases or CNS tumors.

## 1. Introduction

**Figure 1:**
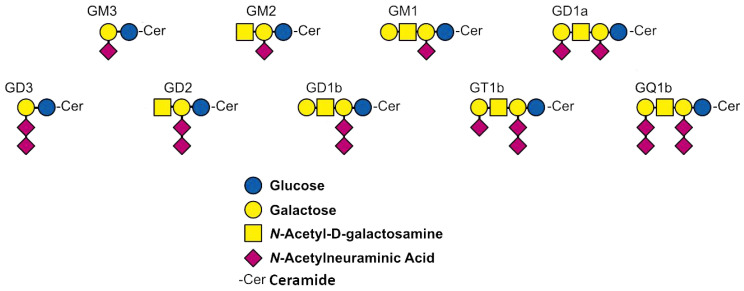
Structure of the nine gangliosides most abundant in biological samples [4].

The discovery of gangliosides is related to the German biochemist Ernst Klenk. In 1942, he identified these N-acetylneuraminic acid-containing glycolipids while studying postmortem brain samples. He named them gangliosides in reference to their location and presumed dominant role in ganglion cells [1,2]. Gangliosides are glycosphingolipids containing one or more sialic acids (Figure 1). They are mainly located in the cell membrane, concentrated in so-called lipid rafts [3]. The hydrophobic ceramide lipid portion is embedded in the outer layer of the plasma membrane, while the hydrophilic glycans extend into the extracellular space.

Despite their prevalence in vertebrate cells and tissues, they are particularly abundant in the central nervous system [5]. Gangliosides account for 10–12% of the lipid content of the nervous system and form cell-type-specific patterns on the surface of neuronal cells that change with differentiation [6]. Ganglioside expression is regulated during development and is tissue and cell-type-specific. In the central nervous system, GM1, GD1a, GD1b, GQ1b, and GT1b are predominantly expressed, whereas cancer cells contain a relatively high amount of the gangliosides GM3, GM2, GD3, and GD2 [7,8]. Although the precise biological functions of gangliosides remain elusive, it is known that despite their relatively low abundance, they stabilize neuronal plasma membranes in association with sphingomyelin and cholesterol. They also play an active role in the formation and dynamics of membrane microdomains [9]. Additionally, they are thought to be involved in brain development, as the pattern of gangliosides changes during this process. Initially, simple GM3 and GD3 gangliosides are predominant, followed by the more complex GD1a and GT1b gangliosides. Furthermore, the ganglioside content and composition of the brain also undergoes changes with aging. The concentration of GQ1b, GT1b, and GD1b increases with age, while that of GM1 and GD1a decreases [10,11]. In addition, gangliosides affect the structure and function of the nervous system, metabolic processes, and the pathomechanism of inflammation [12]. Several studies also suggest that gangliosides play an important role in tumorigenesis. Gangliosides may thus affect functions such as cell adhesion, motility, differentiation, metastasis, and angiogenesis [13]. Given their diverse functions, the development of methods for their analysis is essential to gain better insight into the role of gangliosides in the regulation of physiological and pathological processes. From the 1990s, ganglioside analysis has made significant technological advances and recently, methods suitable for the qualitative and quantitative identification of these components in complex tissue and body fluid samples have been introduced [14]. Initially, ganglioside analysis involved the utilization of thin layer chromatography (TLC) and immunochemical and immunohistochemical methods. Despite their relatively low instrumentation and operation costs, these methods provide information only on the major gangliosides present in the sample because of their low sensitivity [15]. Consequently, modern instrumental techniques with high sensitivity and accuracy, such as mass spectrometry (MS), have been progressively introduced into the field of glycolipid analysis [16,17]. However, chromatographic or electrophoretic separations are typically needed prior to the MS analysis of heterogeneous carbohydrate mixtures. Liquid-based separation methods, such as high-performance liquid chromatography (HPLC) and capillary electrophoresis (CE), have proven to be suitable for the analysis of carbohydrates [18]. Nevertheless, the applicability of liquid chromatography is not without limitations, e.g., the separation of ganglioside isomers, such as GD1a and GD1b [19]. CE is an efficient and versatile method for the separation of complex sample mixtures from biological sources. It is distinguished by low sample requirements and electrolyte consumption. It is a universally applicable technique for the analysis of small molecules, proteins, nucleic acids, and complex glycoconjugates [18]. CE is particularly well-suited for the analysis of glycans, as it can provide both an excellent and rapid separation of positional and linkage isomers, making it suitable for the analysis of the most critical gangliosides [20]. The separation of sugars is based on the difference in their charge-to-hydrodynamic volume ratio, easily recognizing differences in the shape of glycan structures. A common approach in the analysis of glycoproteins is fluorescent labeling of their oligosaccharide moiety released by enzymatic digestion, followed by the analysis of the labeled glycan using capillary electrophoresis with laser-induced fluorescence detection (CE-LIF). Typically, 8-aminopyrene-1,3,6-trisulfonic acid (APTS) is used to label carbohydrates by reductive amination. This labeling method results in derivatives with excellent fluorescence properties and three sulfonic acid groups. By providing negative charges, this latter is useful for electromigration [21]. The method can also be applied for gangliosides. After the removal of the ceramide chain by enzymatic digestion, the glycan moiety is labeled with APTS and analyzed using CE-LIF. Previously, our research group developed a CE-UV method for the separation and quantification of the most abundant neural gangliosides, namely GM1, GD1a, GD1b, GT1b, GQ1b, and the extraneural GM3 [22]. However, because of the short path length and limited selectivity, the sensitivity of UV detection allows the quantification of gangliosides present in the µM concentration range. The objective of the work presented in this article was to develop and validate a more sensitive CE-LIF method for the separation and quantification of the nine most abundant gangliosides in biological samples in their fluorescently labeled glycan form. The developed method is suitable for the separation of the characteristic gangliosides in the central nervous system, GM1, GT1b, GQ1b, and the GD1a and GD1b isomers, as well as the gangliosides GM2, GM3, GD2, and GD3, which may play a major role in malignant processes. In our work, we optimized the sample extraction and separation conditions and validated the required analytical parameters, including linearity, limit of detection (LOD), limit of quantification (LOQ), precision, and accuracy. In addition, we examined the stability of the samples. We demonstrated the applicability of the method in the determination of gangliosides in different cell lines of central nervous system origin. The developed method has great potential in the analysis of ganglioside pattern change in CNS disorders and in tumors.

## 2. Results and Discussion

### 2.1. Optimization of Separation Conditions

In glycan analysis using CE-LIF, weakly acidic background electrolytes containing various polymer additives, e.g., polyethylene oxide (PEO) and polyethylene glycol (PEG), are commonly used [23]. The incorporation of viscosity-enhancing polymers has been demonstrated to markedly enhance the separation of components, particularly in the case of complex glycan samples. The objective of the optimization was to create conditions for the separation of the nine ganglioside glycan standards (GD3, GM3, GD2, GQ1b, GT1b, GM2, GD1a, GD1b, and GM1).

A number of oligosaccharide compounds have been evaluated as potential internal standards, including maltoheptose, maltopentose, maltotriose, and maltose. Maltotriose was selected for its close migration to the range of those of gangliosides under optimal running conditions, not significantly extending the separation time. No interfering substances were observed at the migration time of the IS.

#### 2.1.1. Effect of Buffer Concentration and Different Viscosity Enhancing Compounds on Separation

The zone electrophoretic separation of the labelled glycans of the nine gangliosides was performed in LiAc buffer. The reverse polarity separation mode was used, as labelled glycans have several acidic groups providing negative charges. The concentration of LiAc buffer was tested in the range of 10–30 mM. Without additives, no adequate separation was achieved. The separation of GD1a and GD1b isomers was not observed at any concentration. Migration times increased with rises in the buffer concentration. A fifteen mM LiAc buffer concentration was selected for further optimization through the testing of different buffer additives.

PEO (100 kDa) was initially investigated as a potential viscosity-enhancing compound. The concentration range tested was between 0.1 and 0.4% *w*/*v*. No adequate separation was observed at any of the concentrations used, and the comigration of gangliosides with an excess of labeling reagent was observed. PEG (35 kDa) was then tested in the concentration range of 1 to 5% *w*/*v*. The comigration of the GD1a and GD1b isomers, along with GM2 and GT1b, were found, and several peaks derived from the labeling reagent migrated within the range of the gangliosides. A range from 15 to 25% *v*/*v* glycerol was tested next as a potential buffer additive. The best separation was observed at 17%, although the separation of the critical isomers was incomplete, and migration times were prolonged (Figure S1).

The efficacy of the linear polyacrylamide (LPA, 10 kDa) as a viscosity-enhancing polymer network was also investigated, in the 1% to 7% *w*/*v* concentration range. Although the separation of critical isomers occurred even at the lower concentrations, several gangliosides exhibited comigration, including GM2 with GT1b and GD2 with GQ1b. However, at a 5% LPA concentration, all nine ganglioside glycans were successfully separated. 

The 5% LPA additive is above the entanglement threshold, allowing it to form an effective polymer network [24]. At this concentration, the LPA molecules overlap and intertwine, creating a three-dimensional matrix within the capillary. This entangled polymer network provides a sieving effect, which can explain the significantly improved separation of oligosaccharides.

The sieving effect is of particular importance for the separation of ganglioside isomers-derived glycans, which differ only in the spatial arrangements of their sialic acid groups. The entangled LPA network acts as a molecular sieve, and discrimination between the isomers is likely based on their different shape.

#### 2.1.2. Effect of pH and Buffer Concentration on the Separation in the Presence of 5% *w*/*v* LPA

Following the optimization of the separation buffer additive, the impact of pH on the resolution was evaluated in the range of pH 4.0 to 6.0, with steps of 0.5 pH units, except for around the pKa of acetic acid (pH 4.75), where finer steps of 0.25 pH units were used to gain a more precise understanding. 

It was found that at pH of 4, the baseline separation of GQ1b and GD2, and GT1b and GM2 glycan pairs was not achieved. 

Upon increasing the pH to 4.5, an improvement in the separation of GT1b and GM2 gangliosides was observed. Nevertheless, the separation of GQ1b and GD2 peaks remained unsatisfactory.

**Figure 2:**
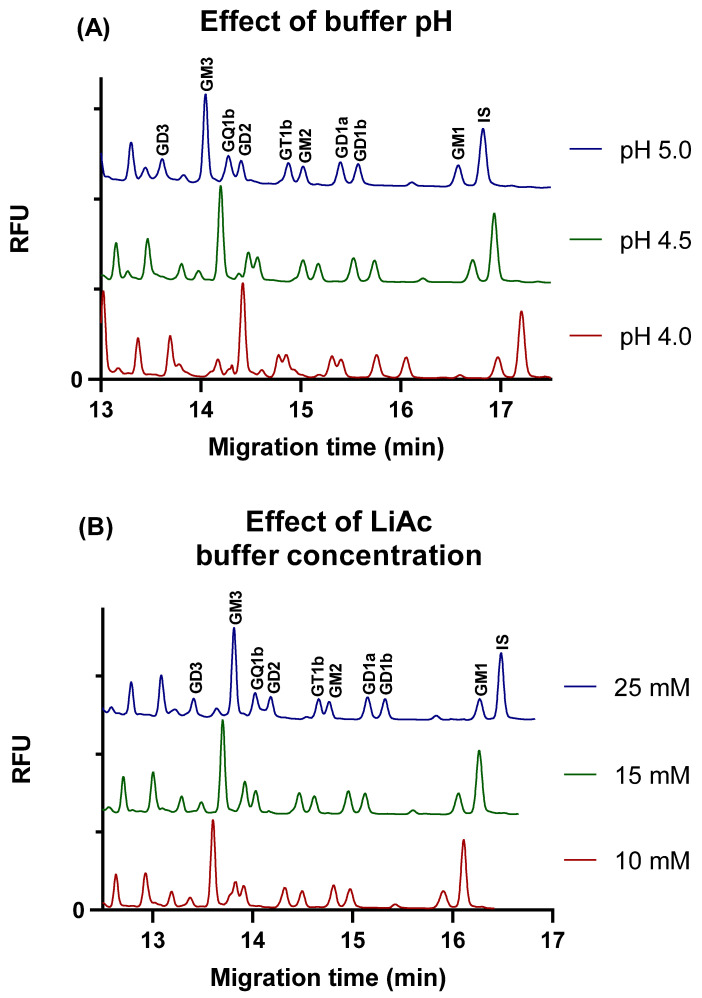
Effect of various buffer pH (**A**) and LiAc buffer concentration (**B**) on the separation of the glycans of nine gangliosides. Separation voltage: −25 kV; temperature: 25 °C; capillary: 50/60 cm × 75 µm i.d. uncoated fused silica capillary; injection 0.5 psi/5 s.

In the pH range of 4.75 to 6, the separation of all nine ganglioside glycans was acceptable. The effect of different buffer pH values on the separation is shown in Figure 2A.

The impact of the LiAc background electrolyte concentration on the separation was meticulously examined at a pH of 5 in the presence of a 5% LPA additive. Throughout the investigation, LiAc concentrations were varied between 10 and 30 mM in incremental steps of 5 mM. Electropherograms illustrating the effect of LiAc concentration are shown in Figure 2B.

At a 10 mM LiAc concentration, the minimal resolution of the peaks of GQ1b and GD2 ganglioside glycans was observed. Increasing the LiAc concentration to a 15 mM resolution of GQ1b and GD2 gangliosides improved significantly. However, a further increase in the buffer concentration had a negative impact on the separation of GT1b and GM2 gangliosides that began to overlap at a concentration of 25 mM and above. Neither pH nor buffer concentration had a significant effect on the resolution of GD1a and GD1b isomers. 

As optimum separation conditions, a 15 mM LiAc buffer with pH of 5.0 was chosen; when both GQ1b and GD2, and GT1b and GM2 gangliosides were sufficiently separated, this enabled the efficient and accurate analysis of the gangliosides.

A small non-selective reduction in migration times was observed with increases in pH and decreases in buffer concentration, which might be explained by the slightly altered viscosity or sieving effect of the polymer matrix.

### 2.2. Optimization of the Digestion

The digestion efficiency was analyzed over a period of 0.5 to 16 h. The samples were analyzed until a plateau was reached in the peak areas, suggesting the completion of the digestion process.

The peak areas exhibited a steep increase between 1 and 8 h, followed by a slower one, and plateaued between 14 and 16 h. This is advantageous for experimental protocols as it ensures that the digestion process can be completed overnight (see the process described in Section 3.3.3).

### 2.3. Optimization of the Glycan Labeling

APTS is the most frequently utilized labeling reagent for glycan analysis using capillary electrophoresis, largely due to practical considerations. The fluorescence intensity of APTS-labeled glycans is 40 times greater than that of unconjugated APTS, and its excitation wavelength fits to the 488 nm Ar-ion laser source of LIF detectors, in contrast to the majority of other labeling reagents [25].

The optimum concentration of APTS was studied in the range of 100 µM to 60 mM. Our results demonstrated a proportional increase in signal intensity with higher concentrations of APTS with a slower rate of intensity enhancement above 40 mM. At concentrations exceeding 50 mM, the emergence of interfering peaks in the electropherograms was observed; therefore, this concentration was chosen for the further experiments. 

The incubation time of the reaction was next optimized across the range from 0.5 to 4 h. The maximum peak intensities were observed after a two-hour incubation period. Subsequently, a decline in peak area was observed, which was particularly evident in the case of GQ1b, containing four sialic acid moieties. This is in line with previous findings of sialic acid loss during a longer incubation at a higher temperature in acidic pH levels [26,27]. Upon the consideration of the aforementioned factors, an incubation period of two hours was identified as the optimal duration.

### 2.4. Method Validation

The optimized method was validated using ganglioside extracts from glioblastoma cells spiked with the ganglioside standards. The linearity range of gangliosides was investigated between 50 and 1250 nM concentrations. Exceptions are the gangliosides GM3 and GQ1b. For GM3, the concentration range was selected between 500 and 2500 nM, while for GQ1b, the concentration range was selected within the range of 25 to 625 nM.

**Figure 3:**
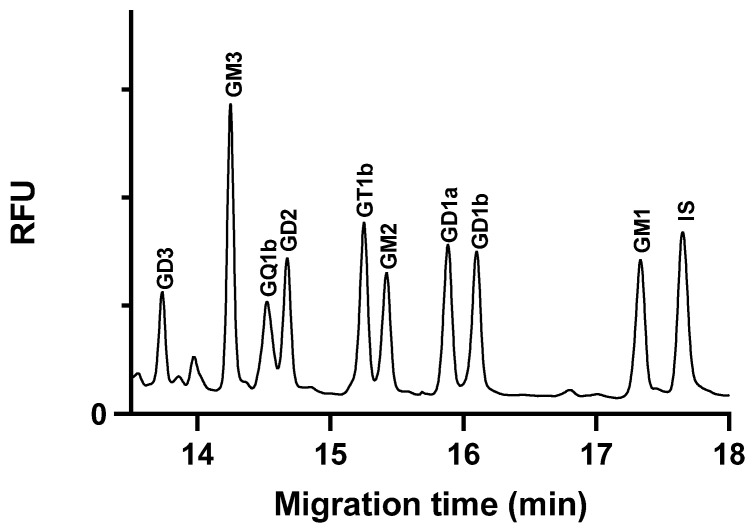
Glycan separation of ganglioside spiked glioblastoma cell extracts under optimized separation conditions. The cells were spiked with 1250 nM ganglioside standards each, except GQ1b, which was 625 nM. Separation buffer: 15 mM LiAc, pH 5.0, containing 5% *w*/*v* LPA. Separation voltage: −25 kV; temperature: 25 °C; capillary: 50/60 cm × 75 µm i.d. uncoated fused silica capillary; injection 0.5 psi/5 s.

The calibration concentration range was selected based on the anticipated ganglioside content of the samples, which is expected to fall within the specified range. The high concentration range for the GM3 ganglioside was justified by its high concentration in the C6 glioblastoma cells. An internal standard was used to normalize the peak area values. Figure 3 illustrates the electropherogram of a spiked cell extract sample.

As illustrated in Table 1, the correlation coefficient values for the six-point calibration curves are between 0.9914 to 0.9993, thereby confirming a linear relationship between peak area ratios and concentrations within the calibration range for all nine gangliosides. 

Given that gangliosides are endogenous compounds, it was not possible to obtain an analyte-free biological sample as the matrix for calibration purposes. In lieu of an analyte-free biological sample, analyte-spiked samples were utilized to construct the calibration curves, which were subsequently subjected to background subtraction (see in Section 3.4.1). The calculated LOQ and LOD values from the calibration curves ranged from 24.7 to 103.7 nM and 8.1 to 34.2 nM, respectively. An exception was observed for GM3, where the LOQ and LOD values were higher, 208.5 nM and 68.8 nM. This ganglioside was present in the highest concentration in the cell extracts that served as the sample matrix. It is important to note that the measurement of the baseline level of gangliosides in the blank biological samples contributes to the standard deviation of the calibration samples. Consequently, the relatively high LOQ and LOD values do not necessarily reflect the true analytical sensitivity of the CE method, and lower concentrations can indeed be detected.

In comparison to our previously published CE-UV method, which achieved detection limits in the µM range [22], the CE-LIF method developed and applied in this study allows the quantification of gangliosides in the nM range. The sensitivity is comparable to that of LC-MS techniques, which are also reported in the nM range [28,29].

Intraday and interday accuracy and precision (see in Section 3.4.2) were evaluated using quality control (QC) samples at three concentration levels and were found within the acceptable limits (Table 2). This indicates that the method produces consistent, reliable, and repeatable results across different days, confirming its applicability for routine analytical use.

In this work, extraction recovery values were not investigated separately, as the same validated liquid–liquid extraction procedure was used as in our previously published study [22]. The extraction recovery values previously studied for analytes measured at low, medium, and high concentrations were higher than 84% with an RSD of less than 13%.

The samples stored at 5 °C demonstrated stability for a period of 24 h, with accuracy values ranging from 86% to 114% and RSDs below 14%. For the samples stored at −20 °C for 1 week, the accuracy values were between 85% and 106%, with RSDs under 12%. These results are within acceptable limits, indicating appropriate stability.

### 2.5. Application of the Method

**Figure 4:**
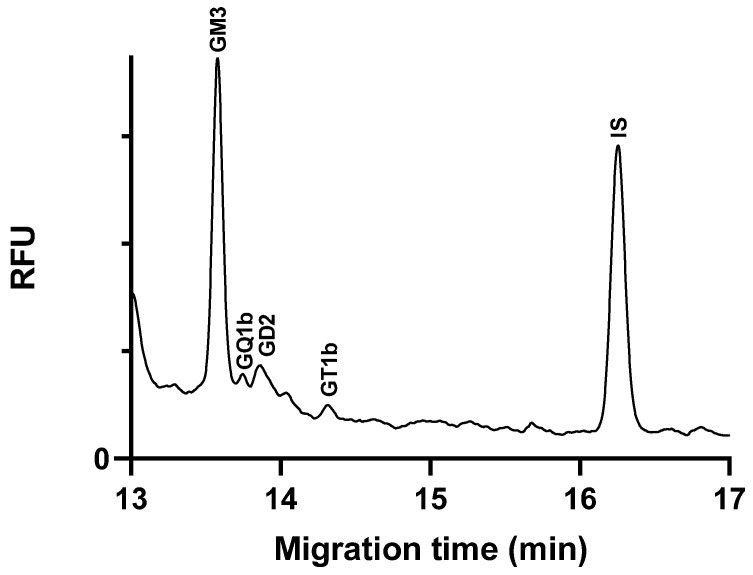
Electropherogram of ganglioside extract glycans from C6 glioblastoma cell line sample. Separation buffer: 15 mM LiAc, pH 5.0, containing 5% *w*/*v* LPA. Separation voltage: −25 kV; temperature: 25 °C; capillary: 50/60 cm × 75 µm i.d. uncoated fused silica capillary; injection 0.5 psi/5 s.

To demonstrate the applicability of the method, we analyzed, quantified, and compared the ganglioside content in two cell lines of central nervous system origin. The peak identification was performed by spiking with standards, and the amounts of gangliosides were normalized to the protein content of the samples (see in Section 3.3.2) and are given as nmol/mg protein to ensure comparability. Figure 4 shows the electropherogram of a glioblastoma cell line sample.

Gangliosides GM3, GD2, GT1b, and GQ1b were identified in glioblastoma cells (Table 3). Among these, GM3 was found to be present in the highest amount, consistent with the literature reports indicating elevated GM3 levels in glioblastoma tumors [30]. The analyses were conducted when the cells had reached at least 80% confluency. The literature data indicate that the expression and content of the GM3 ganglioside in C6 glioma cells vary significantly depending on the cell density. Sparse and confluent cells show different levels of GM3, with the expression increasing as the cells become more confluent [31]. In our study, the high GM3 levels observed in the glioblastoma cell samples can be attributed to the high cell density at the time of analysis. This correlation between cell density and GM3 content highlights the importance of considering cell confluency in studies involving ganglioside analysis. It also underscores the relevance of GM3 as a biomarker for glioblastoma, given its consistent elevation in the high-density tumor cell line.

**Figure 5:**
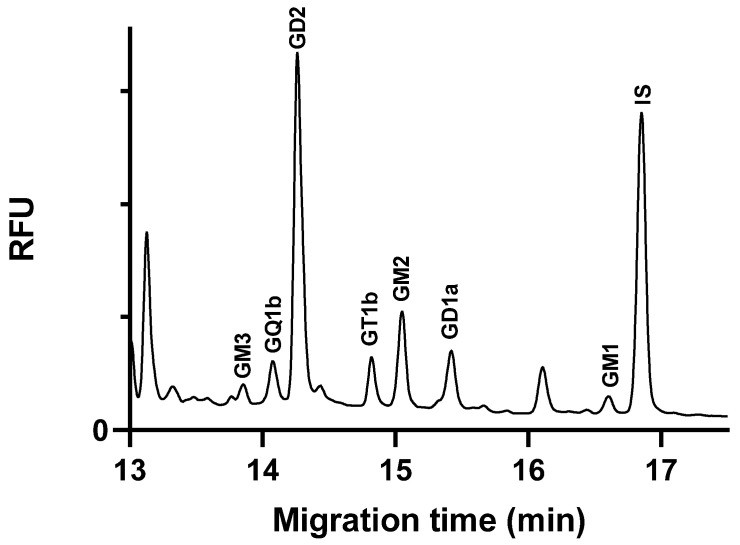
Electropherogram of ganglioside extract glycans from SH-SY5Y neuroblastoma cell line sample. Separation buffer: 15 mM LiAc, pH 5.0, containing 5% *w*/*v* LPA. Separation voltage: −25 kV; temperature: 25 °C; capillary: 50/60 cm × 75 µm i.d. uncoated fused silica capillary; injection 0.5 psi/5 s.

In the neuroblastoma cells, we identified several central nervous system (CNS)-specific gangliosides, including GM1, GD1a, GT1b, and GQ1b (Figure 5). These gangliosides are critical components of neuronal cell membranes and play important roles in cell signaling and interaction [4]. In addition, we detected tumor-associated gangliosides in the samples, predominantly GD2, and lesser amounts of GM2 and GM3.

The elevated levels of GD2 in neuroblastoma cells are consistent with the literature, which contains extensive discussions about the strong association between the GD2 ganglioside and neuroblastoma [32]. GD2 is highly expressed on the surface of neuroblastoma cells, but its presence is minimal in normal tissue cells, making it an excellent therapeutic target for this type of cancer [33]. The anti-GD2 monoclonal antibody, dinutuximab, is approved for the treatment of high-risk childhood neuroblastoma [34].

Overall, our method has successfully identified and quantified gangliosides in cell lines of central nervous system origin and the results are consistent with the literature data.

## 3. Materials and Methods

### 3.1. Chemicals

All reagents and chemicals were at least of analytical grade purity. APTS was supplied by Apollo Scientific (Stockport, UK). Sodium cyanoborohydride, polyethylene oxide (1000 kDa), polyethylene glycol (35 kDa), and lithium acetate were provided by Thermo Fischer Scientific (Waltham, MA, USA). Methanol, chloroform, acetic acid, and hydrochloric acid solution were obtained from Molar Chemicals Ltd. (Halásztelek, Hungary). Linear polyacrylamide solution (50%, 10 kDa) was from Polysciences (Warrington, PA, USA). Glycerol and antibiotic solutions were procured from the University Pharmacy of Semmelweis University (Budapest, Hungary). Sodium hydroxide (NaOH), sodium acetate, ganglioside standard GD2, tetrahydrofuran, Triton X-100, and maltotriose (used as an internal standard, IS) were purchased from Merck (Darmstadt, Germany). Ganglioside standards GM3, GM2, GM1, GD1a, GD1b, GD3, GT1b, and GQ1b were obtained from Cayman Chemical (Ann Arbor, MI, USA). Recombinant endoglyceramidase I (EGC-ase I) enzyme, produced in *Rhodococcus erythropolis*, was sourced from TCI Chemicals (Tokyo, Japan). Fetal bovine serum (FBS) was purchased from Biosera (Nuaille, France). Dulbecco’s Modified Eagle Medium (DMEM) and trypsine-EDTA solution were produced by Corning (Tewksbury, MA, USA). Ultrapure water was obtained from a Millipore Direct-Q 3UV System (Merck).

### 3.2. Instrumentation

Measurements were conducted using a 32 Karat 5.0 software-controlled P/ACE MDQ (Beckman Coulter Inc., Brea, CA, USA) capillary electrophoresis instrument with a LIF detector equipped with an Argon ion laser. Fluorescence was detected at 488 nm excitation and 520 nm emission wavelengths. Uncoated fused silica capillaries (Agilent Technologies, Santa Clara, CA, USA) with an internal diameter of 75 µm and an outer diameter of 365 µm were applied. The effective and total capillary lengths were 50 cm and 60 cm, respectively. The initial conditioning of the capillary was achieved through the flushing of the capillary with a 1 M NaOH solution (60 psi, 10 min), a 0.1 M NaOH solution (60 psi, 10 min), and then ultrapure water (60 psi, 25 min). Capillary was flushed with 1 M HCl (60 psi, 3 min), ultrapure water (60 psi, 3 min), and separation buffer (60 psi, 3 min) between each run. Prior to use, separation buffers were degassed for a period of five minutes in an ultrasonic bath. The autosampler unit was maintained at 5 °C, and the measurements were carried out at a constant temperature of 25 °C. A constant voltage of −25 kV (−416 V/cm) was applied during the separation process. Reverse polarity separation mode was used. Samples were injected into the capillary using hydrodynamic injection (0.5 psi, 5 s, corresponding to approximately 20 nL of sample volume). The identification of gangliosides was conducted by spiking samples with analytical standards.

### 3.3. Sample Preparation

#### 3.3.1. Preparation of Cells

C6 rat glioblastoma and SH-SY5Y neuroblastoma cells were cultured in DMEM containing 2 mM stable glutamine and 1 mM pyruvate and supplemented with 10% FBS, 80 µg/mL gentamicin, and 10 µg/mL ciprofloxacin. Cells were cultured at 37 °C in 5% CO_2_/95% humidified air. Adherent cells were removed from the petri dishes after reaching at least 80% confluency using trypsin-EDTA solution, collected in a centrifuge tube, and centrifuged at 500× *g* for 5 min. The supernatant was then removed, and the cells were resuspended in 1 mL PBS and centrifuged at 20,000× *g* for 10 min.

#### 3.3.2. Ganglioside Extraction

The gangliosides were extracted using a liquid–liquid extraction method as described by Svennerholm and Fredman [35], with slight modifications [22]. The cells collected by centrifugation were washed twice with approximately 10 volumes of PBS to remove any residual contaminants. Following removal of PBS, 10 mg wet weight of cells were homogenized in 3 volumes of water by using an ultrasonic homogenizer (Labsonic 2000, B. Braun, Melsungen, Germany). The protein content of the homogenized sample was determined by the Bradford method [36]. To the prepared homogenate, a solution of methanol (10.6 volumes) and chloroform (5.3 volumes) containing ganglioside standards was added. The resulting mixture was gently vortexed and then centrifuged at 1000× *g* for 8 min. The supernatant was collected, and the cell pellet was re-extracted with a chloroform–methanol–water mixture (4:8:3, *v*/*v*/*v*). The total lipid extract was obtained by combining the supernatants. Subsequently, water (0.173 volume) was added to the lipid extract, creating a hydrophilic upper phase (U1) and a hydrophobic lower phase. The hydrophilic upper phase (U1) was collected, and the hydrophobic lower phase was re-extracted with a theoretical upper phase of the same volume as U1. The resulting upper phase (U2) was combined with U1, and the combined upper phase was then re-extracted with the theoretical lower phase. Subsequently, the aqueous phase containing the gangliosides was evaporated to dryness under a nitrogen stream (Zymark TurboVap LV, Hopkinton, MA, USA) in a water bath at 37 °C and stored at −20 °C until required.

#### 3.3.3. Digestion of Gangliosides by Endoglycoceramidase

Endoglycoceramidase I (EGCase I) enzyme was employed to cleave glycan groups from gangliosides. The reaction was conducted in a buffer comprising 50 mM sodium acetate at a pH of 5.5 and 0.1% Triton X-100, which proved to be the optimal conditions for the enzyme reaction. To prepare the reaction mixture, 0.06 mU of EGCase I enzyme was added to 2 nmol of ganglioside standard, and the samples were incubated at 37 °C for sixteen hours. The tubes were then placed in ice cold water to halt the reaction. The hydrolyzed samples were then evaporated under a nitrogen stream in a water bath at 37 °C and used for derivatization. For biological samples, an aqueous solution of ganglioside extract equivalent to 10 mg of cells was subjected to enzymatic digestion using the same procedure.

#### 3.3.4. Fluorescence Derivatization

Glycans of the ganglioside standards were labelled with APTS. For derivatization, 2 nmol of the evaporated glycan sample from the ganglioside standard was dissolved in 2 μL of water and 10 μL of labeling reagent was added. The composition of labeling reagent was as follows: 4 μL of 20% acetic acid, 2 μL of 1 M Na-cyanoborohydride dissolved in tetrahydrofuran, and 4 μL of 50 mM APTS dissolved in acetic acid. The reaction mixture was incubated in a water bath at 37 °C for 2 h and then evaporated to dryness under a stream of nitrogen. The evaporated labelled glycans were stored at −20 °C until further use. For CE measurements, maltotriose was used as an internal standard and labelled in the same way as ganglioside glycans. Stock solutions of the fluorescently labelled evaporated glycan standard samples were prepared by dissolution in 100 μL water. The derivatization of ganglioside glycans obtained from 10 mg cells was performed in accordance with the standard procedure for the glycan labelling. Stock solutions were prepared by adding 50 μL of water to the evaporated labelled glycans.

### 3.4. Method Validation

#### 3.4.1. Calibration Curves

To accurately quantify gangliosides, a six-point calibration curve was prepared to determine the linear range. Ganglioside extracts from C6 glioblastoma cells were used as a matrix for the preparation of calibrators, spiked with known concentrations of ganglioside standards and IS before extraction, followed by the sample preparation steps described previously. The background subtraction method was used to obtain the calibration points, which is one of the recommended methods for the validation of endogenous compounds in the official ICH guideline M10 [37]. Blank samples spiked with the IS were used to determine the baseline levels of the analytes in the matrix, which were subtracted from the responses of the spiked calibration samples. The peak area ratios of the analytes and the IS were employed in the calculations.

The LOQ and LOD values were calculated in accordance with the ICH Q2(R1) guideline [38] employing the following equations:

LOQ: 10σ/S(1)

LOD: 3.3σ/S(2)

where σ represents the standard deviation of the Y-intercept, and S denotes the slope of the calibration curve.

#### 3.4.2. Precision and Accuracy

Intraday and interday accuracy and precision across the quantification range were evaluated using QC samples, each analyzed in quadruplicate. The evaluations were conducted over three separate days, with three independent runs performed each day. As with the calibration samples, ganglioside extracts from spiked cells were employed as QC samples. QC samples were prepared at three distinct concentration levels. The precision was deemed acceptable within ±15% RSD, except for the lowest QC level, where ±20% was considered permissible. The accuracy was regarded as acceptable within ±15% of the nominal concentration, with ±20% being tolerated at the lowest QC level.

#### 3.4.3. Stability of the Extracted Sample

The stability of the QC samples was investigated in four replicates at three different concentration levels under a variety of conditions. Evaporated glycan samples dissolved in water were stored for 24 h at 5 °C and then analyzed to evaluate the autosampler stability. For long-term stability testing, prepared samples were stored at −20 °C for 1 week. A deviation of less than ±15% from the nominal concentration was deemed acceptable.

### 3.5. Statistical Analysis

The statistical analysis was conducted using GraphPad Prism 8 (GraphPad Software, La Jolla, CA, USA). A weighted regression analysis was conducted using 1/y weights to assess the calibration curve.

## 4. Conclusions

A validated CE-LIF method was successfully developed for the separation and quantification of the glycans of nine gangliosides. By optimizing the sample preparation and separation conditions, a reliable method was elaborated, characterized by high sensitivity, accuracy, and precision. The LOQ and LOD values are comparable to those of LC-MS techniques.

This optimized method was applied to the analysis of gangliosides in glioblastoma and neuroblastoma cell lines, respectively. In glioblastoma cells, GM3 was identified as the predominant ganglioside, which is in line with the literature reports of elevated GM3 levels in this type of tumor. Furthermore, we identified and quantified GD2, GT1b, and GQ1b in the cells in a lower amount. In neuroblastoma cells, we identified CNS-specific gangliosides such as GM1, GD1a, GT1b, and GQ1b, as well as tumor-associated gangliosides, predominantly GD2, and smaller amounts of GM2 and GM3. The high GD2 level corroborates the existing research and highlights its importance as a drug target in neuroblastoma therapy.

The presented CE-LIF method represents a reliable, sensitive, and efficient approach for the separation and quantification of gangliosides in biological samples. It holds significant potential for further studies on ganglioside patterns in CNS disorders and tumorigenesis, offering valuable insights into their role in various physiological and pathological processes.

## Figures and Tables

**Table 1:** Validation parameters of linearity, LOQ, and LOD of the optimized method.

	Linear Range (nM)	Slope	Y-Intercept	Std. Error of Slope	Std. Error of Y-Intercept	Correlation Coefficient (R^2^)	LOQ (nM)	LOD (nM)
GM1	125–1250	7.58 × 10^−4^	−2.31 × 10^−2^	3.30 × 10^−5^	6.72 × 10^−3^	0.9944	88.7	29.2
GM2	125–1250	5.84 × 10^−4^	−1.78 × 10^−2^	9.81 × 10^−6^	2.00 × 10^−3^	0.9992	34.2	11.3
GM3	500–2500	7.18 × 10^−4^	−1.23 × 10^−1^	2.51 × 10^−5^	1.50 × 10^−2^	0.9963	208.5	68.8
GD1a	125–1250	7.36 × 10^−4^	−3.66 × 10^−2^	3.09 × 10^−5^	1.82 × 10^−3^	0.9930	24.7	8.1
GD1b	125–1250	8.10 × 10^−4^	−6.80 × 10^−2^	1.96 × 10^−5^	6.08 × 10^−3^	0.9982	75.1	24.7
GD2	125–1250	5.59 × 10^−4^	−8.34 × 10^−2^	2.04 × 10^−5^	5.12 × 10^−3^	0.9960	91.7	30.2
GD3	125–1250	3.36 × 10^−4^	7.01 × 10^−3^	4.59 × 10^−6^	1.40 × 10^−3^	0.9993	41.6	13.7
GT1b	125–1250	7.48 × 10^−4^	−5.01 × 10^−2^	4.02 × 10^−5^	7.76 × 10^−3^	0.9914	103.7	34.2
GQ1b	62.5–625	4.00 × 10^−4^	−3.44 × 10^−3^	1.56 × 10^−5^	1.91 × 10^−3^	0.9940	47.7	15.7

**Table 2:** Intraday and interday precision and accuracy values of the optimized method.

		Intradays	*n* = 4		Interdays	*n* = 4	
	Added Concentration (nM)	Mean (nM)	Accuracy (%)	Precision (RSD%)	Mean (nM)	Accuracy (%)	Precision (RSD%)
	1000	1088.7	108.8	6.6	1038.2	103.8	9.2
GM1	500	498.02	99.6	1.5	489.7	97.9	2.8
	125	132.9	106.3	6.0	112.1	89.7	9.8
	1000	1002.5	100.2	4.2	1066.9	106.6	12.7
GM2	500	497.3	99.4	1.5	555.9	111.2	5.6
	125	107.2	85.7	7.8	112.3	89.8	13.3
	2000	2221.2	111.0	2.1	2295.8	114.7	5.9
GM3	1250	1070.9	85.6	8.1	1275.1	102.0	4.1
	500	545.3	109.0	4.6	562.8	112.5	14.6
	1000	1041.3	104.1	7.6	1131.9	113.2	7.9
GD1a	500	493.7	98.7	4.1	564.6	112.9	3.2
	125	134.0	107.2	3.5	135.7	108.6	7.9
	1000	1063.8	106.3	9.7	1062.1	106.2	4.4
GD1b	500	450.4	90.0	4.8	501.5	100.3	3.9
	125	122.5	98.0	14.8	119.7	95.7	10.3
	1000	996.1	99.6	8.6	1098.7	109.8	14.1
GD2	500	464.6	92.9	5.6	474.0	94.8	11.7
	125	117.6	94.0	11.7	130.6	104.5	10.3
	1000	1054.7	105.4	8.1	1123.0	112.3	4.2
GD3	500	495.1	99.0	7.4	480.1	96.0	5.5
	125	119.8	95.9	5.5	121.4	97.1	13.3
	1000	1088.0	108.8	1.1	1093.6	109.3	8.6
GT1b	500	443.9	88.7	9.6	528.2	105.6	10.2
	125	111.4	89.1	8.8	130.2	104.2	8.1
	500	496.0	99.2	8.1	537.4	107.5	12.8
GQ1b	250	268.7	107.5	4.6	276.9	110.7	8.9
	62.5	58.4	93.5	7.2	73.7	117.9	13.2

**Table 3:** Ganglioside content (nmol/mg protein) measured in the two cell lines. The values are means ± SD (*n*  =  4).

**Ganglioside Content (nmol/mg Protein)**	
C6 Glioblastoma	
GM3	20.20 ± 1.97
GQ1b	1.27 ± 0.32
GD2	3.92 ± 0.04
GT1b	1.88 ± 0.14
SH-SY5Y Neuroblastoma	
GM3	2.93 ± 0.28
GQ1b	3.42 ± 0.19
GD2	24.60 ± 3.60
GT1b	2.15 ± 0.38
GM2	6.77 ± 1.37
GD1a	3.60 ± 0.54
GM1	1.30 ± 0.22

## Data Availability

The original contributions presented in the study are included in the article/Supplementary Materials; further inquiries can be directed to the corresponding author.

## Supplementary Materials

The following supporting information can be downloaded at: https://www.mdpi.com/article/10.3390/molecules29163769/s1, Figure S1: Separation of standard mixture of gangliosides with 15 mM LiAc buffer, pH 4.75, containing 17% glycerol additive. Separation voltage: −25 kV.
