# Supplementary material for: Capillary Electrophoresis-Laser Induced Fluorescence Method Development and Validation for Quantification of Nine Gangliosides—Application to Analysis of Cell Lines of CNS Origin
Source: Molecules. 2024 Aug 9;29(16):3769. doi: 10.3390/molecules29163769 (PMC11356799; doi:10.3390/molecules29163769)
Supplement: Supplementary file 1 [file molecules-29-03769-s001.zip › molecules-3129044-supplementary.pdf]

Supplementary

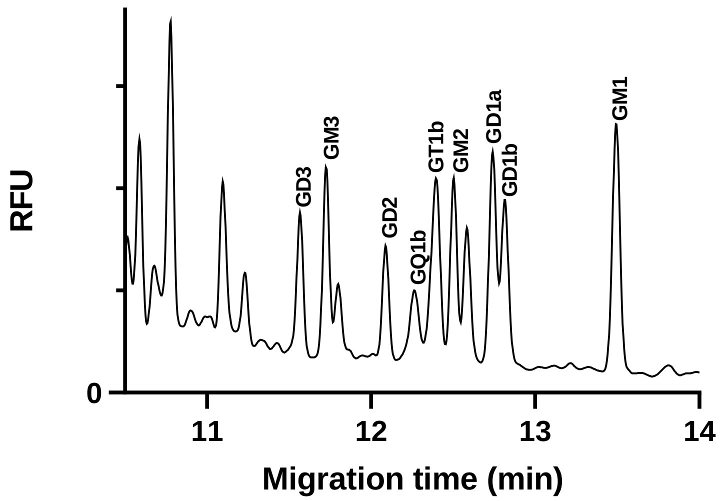

Figure S1: Separation of standard mixture of gangliosides with15 mM LiAc buffer, pH 4.75, containing 17% glycerol additive. Separation voltage: -25 kV.
